# Spatial patterns and key driving factors of wheat harvest index under irrigation and rainfed conditions in arid regions

**DOI:** 10.3389/fpls.2025.1614204

**Published:** 2025-06-09

**Authors:** Yongyu Chen, Hengbati Wutanbieke, Dongdong Zhong, Jian Chen, Zhen Huo, Hegan Dong

**Affiliations:** ^1^ College of Life Sciences, Shihezi University, Shihezi, Xinjiang, China; ^2^ Xinjiang Production and Construction Corps Key Laboratory of Oasis Town and Mountain, Basin System Ecology, Shihezi University, Shihezi, Xinjiang, China; ^3^ Department of Agriculture and Rural Affairs of Xinjiang, Wulumuqi, Xinjiang, China

**Keywords:** arid region wheat, harvest index (HI), irrigation, spatial heterogeneity, driving factors

## Abstract

**Introduction:**

The harvest index (HI), a crucial agronomic trait that measures the ratio of grain yield to aboveground biomass, serves not only as a vital indicator for assessing wheat yield but also as a core parameter for predicting straw resource. It reflects the "source-sink" relationship and biomass allocation strategies in crops. However, the spatial distribution patterns of wheat HI and their key driving factors in arid regions remain unclear.

**Methods:**

This study was conducted in Xinjiang, a typical arid region of China, during 2022–2023, involving two years of large-scale systematic sampling. By integrating multidimensional factors such as geographical and climatic conditions, agronomic management practices, and soil nutrient status, methods including correlation analysis, random forest models, structural equation modeling, and linear regression analysis were employed to systematically investigate the spatial distribution characteristics and driving mechanisms of wheat HI under different irrigation regimes in arid regions.

**Results:**

The results revealed that: (1) Wheat HI in arid regions exhibited significant spatial heterogeneity (0.43–0.67), with an overall distribution pattern of "central high, peripheral low" and "northern high, southern low." (2) The importance rankings of influencing factors differed between irrigation regimes. For irrigated wheat, the order of importance was: Geographic-climatic factors, soil nutrient factors, agronomic management factors. Comprehensive analysis identified longitude (lon), plant height (H), latitude (lat), and bulk density (BD) as the key drivers of the Harvest Index (HI) in irrigated wheat. In contrast, for rainfed wheat, the order was: soil nutrient factors, Geographic-climatic factors, agronomic management factors, with total nitrogen (TN), available phosphorus(AP), total potassium(TK), and total phosphorus (TP) emerging as critical drivers of HI.

**Discussion:**

Irrigation significantly enhanced wheat HI (p < 0.01), and irrigated wheat demonstrated significantly higher HI, yield, and aboveground biomass (AGB) compared to rainfed wheat (p < 0.01). Optimizing phosphorus management could enhance HI in both systems, while irrigation infrastructure development remains vital for yield stability. This study provides a theoretical basis and practical guidance for the synergistic multi-objective approach of “yield increase-irrigation-sustainability” in arid regions wheat production.

## 1 Introduction

Wheat, one of the world’s most essential staple crops, provides approximately 20% of the global dietary calories and protein (Ding et al., 2023; Nouraei et al., 2024) and is hailed as the “king of cereals”. According to statistics from the Food and Agriculture Organization (FAO), global wheat cultivation reached 242 million hectares in 2020, accounting for 34% of the total grain crop area (https://www.fao.org/home/en/). China, the world’s largest wheat producer and fertilizer consumer (Bingjun et al., 2018), contributes 25% of the national grain crop area and 21% of the total yield (National Bureau of Statistics of China, 2022. https://www.stats.gov.cn/english/). However, over the past few decades, the reliance on nitrogen fertilizers to boost yields has led to severe environmental issues (Fan et al., 2012; Huang et al., 2015). In arid and semi-arid regions (Liu et al., 2015), wheat production faces a dual challenge: water scarcity and underutilized straw resources. Due to insufficient and unevenly distributed rainfall, most agricultural areas depend on irrigation, with only a few regions practicing rainfed farming. Although wheat straw is a valuable biomass resource (Koul et al., 2022), its low utilization rate and widespread field burning contribute to severe air pollution (Chen et al., 2013). Notably, the Harvest Index (HI), a key metric for assessing the grain-to-straw ratio, not only influences yield but also directly determines straw biomass availability.

The Harvest Index (HI), defined as the ratio of grain yield to aboveground biomass, has been one of the core drivers of crop yield improvement since the 20th century (Hay, 1995; Sinclair, 1998; Dwivedi et al., 2023). Its biological essence reflects the allocation efficiency of photosynthetic products between “source and sink” (Sinclair, 1998), serving as both a direct indicator for evaluating grain production potential and a key parameter for predicting straw yield (Dai et al., 2016). Studies have shown that the HI of major cereal crops increased significantly during the latter half of the 20th century, contributing to over 50% of global yield growth (Donald and Hamblin, 1976; Hay, 1995). However, the HI of modern high-yielding varieties has approached its biological upper limit (0.4–0.6) (Foulkes et al., 2011; Pradhan et al., 2019) and exhibits significant regional variations. For example, wheat HI in high-rainfall regions of Australia is generally lower than in comparable climatic zones in Europe (Zhang et al., 2012), while interannual environmental fluctuations further contribute to spatial heterogeneity in HI (Ali et al., 2022). In arid regions, optimizing HI is crucial for accurate yield estimation and straw biomass assessment. However, few studies have investigated the distribution patterns of wheat HI under different irrigation regimes in these areas. Therefore, analyzing its spatial distribution characteristics and driving factors will provide a scientific basis for sustainable dryland agriculture and efficient resource utilization.

The wheat harvest index (HI) is regulated by multiple factors, including environmental conditions, irrigation regimes, and soil nutrient status (Du et al., 2024). Research indicates that wheat’s morphological characteristics (e.g., plant architecture, spike type) and physiological traits (e.g., photosynthetic efficiency, assimilate translocation) significantly influence HI (Zhang et al., 2012). Additionally, HI depends on the interaction effects between environment (E) and management practices (M) (Wang et al., 2020). As the ratio of grain yield to aboveground biomass, HI not only reflects the “source-sink” balance but also characterizes the allocation efficiency of photosynthetic products (Li et al., 2022). This allocation pattern is closely associated with crop organ morphology, physiological functions, and stress resistance, representing a crucial adaptive strategy for plants in harsh environments. In agricultural production, improving HI has become a core approach for optimizing water and nitrogen use efficiency while increasing yields (Bilandžija et al., 2023). The high-yield performance of newly developed wheat cultivars in China benefits from the synergistic improvement of high aboveground biomass (AGB) and HI. Modern varieties demonstrate a significant positive correlation between grain yield and biomass (r > 0.8, *p* < 0.01), further validating this mechanism (Huang et al., 2023). As a key yield-determining indicator under drought conditions, HI plays a critical role in crop stress adaptation (Passioura, 1977; Dwivedi et al., 2023). In dryland farming systems, soil nutrient deficiency constitutes a major constraint for crop production (Sietz et al., 2011). With arid regions covering 41% of global land area, their agricultural output is vital for worldwide food security (Liu et al., 2015). Therefore, research on factors influencing wheat HI in arid regions represents not only a frontier issue in agroecological science but also a crucial breakthrough for achieving multiple objectives, including food security, resource conservation, and ecological protection.

Within crop cultivation systems, irrigation serves as a pivotal agronomic practice that exerts decisive influence on yield formation (Herwaarden et al., 1998; Li et al., 2022). This effect is particularly evident in its impact on the harvest index (HI), which has become an ideal breeding selection criterion due to its more stable genetic characteristics compared to yield per se (Donald and Hamblin, 1976; Araus et al., 2002). Research demonstrates that optimized irrigation management can significantly enhance HI under drought conditions (Anantha et al., 2016), with water regulation during the grain-filling period being particularly crucial-moderate water stress promotes remobilization of stem-reserved carbohydrates (Slewinski, 2012), while integrated water-nutrient management achieves synergistic improvement of both yield and resource use efficiency by modulating source-sink relationships (Li et al., 2009). Specifically, water-saving irrigation techniques can increase wheat HI by 15-20% through reducing ineffective tillers and optimizing dry matter translocation (Huang et al., 2023). This finding holds particular significance for dryland agriculture, where water stress frequently causes substantial yield fluctuations (Sietz et al., 2011) and even threatens food security by impairing physiological activity (Abd El-Aty et al., 2022; Christian et al., 2023). Crops adapt through biomass allocation adjustments-for instance, wheat enhances water acquisition capacity by increasing root biomass (Kobe et al., 2010). This regulatory mechanism partially explains the enormous global variation in wheat yields, ranging from <1 t/ha under water- and nutrient-limited conditions to >10 t/ha in favorable environments (Asseng et al., 2020).

However, simply calculating total yield by multiplying cultivated area by yield per unit area may lead to overestimation (Hu et al., 2024). According to U.S. statistics from 1970 to 2017, the final harvested area typically represents less than 85% of the planted area (Zhu and Burney, 2021). Consequently, wheat harvest index (HI) estimates derived solely through this calculation method exhibit substantial errors. This study selects Xinjiang, a representative arid region, as the research area. We conducted extensive small-scale precision sampling across 11 typical wheat-growing counties/cities, directly measuring yield and aboveground biomass per unit area to accurately calculate wheat HI, thereby significantly reducing computational errors. Furthermore, our research incorporates a comprehensive analysis of 19 factors, including: 4 geoclimatic factors, 4 agronomic management factors and 11 soil nutrient factors, to identify the key drivers of wheat HI under different irrigation regimes in arid regions. Specifically, we aim to address two core research questions: (1) What are the specific values and spatial distribution characteristics of wheat HI in arid regions? (2) What are the key driving factors influencing HI variation in these areas?

## 2 Materials and methods

### 2.1 Study area overview

**Figure 1:**
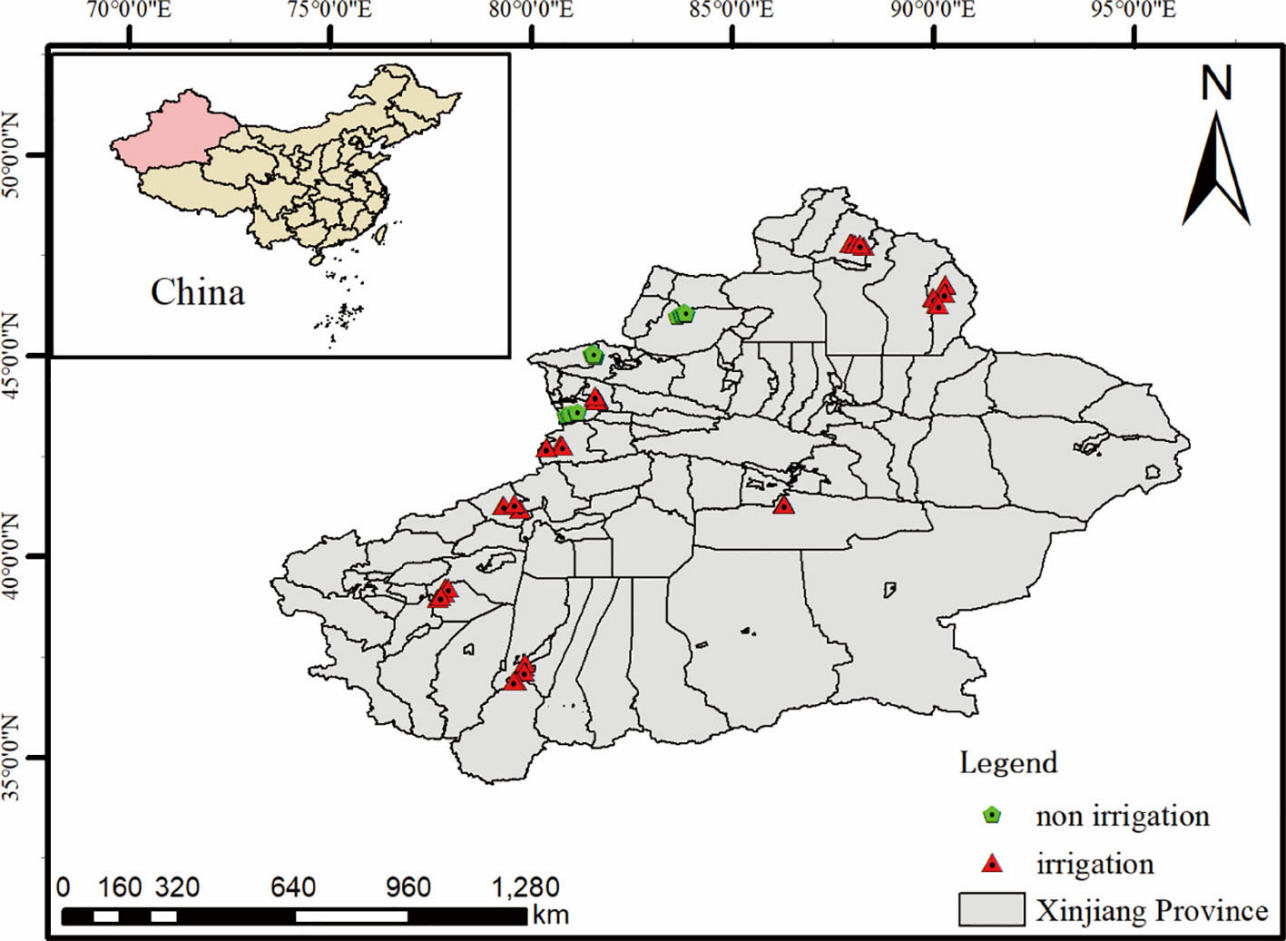
Overview map of the study area and wheat sampling points.

Xinjiang Province is located in the central Eurasian continent of northwestern China, spanning 73°40′–96°18′ E longitude and 34°25′–48°10′ N latitude (**Figure 1**). With a total area of 1.66 million km², it constitutes the largest administrative region in China. The topography features alternating mountain ranges and basins, where basins are encircled by high mountains, forming a distinctive “three mountains flanking two basins” geomorphological pattern that creates a unique “irrigation agriculture and oasis economy” system.

As an inland region far from oceans, Xinjiang exhibits pronounced continental climate characteristics. It has a typical temperate continental arid climate with scarce precipitation (annual mean: 50–200 mm) and strong evaporation. The annual average temperature ranges from 4–13°C, with a frost-free period of 130–220 days. These conditions provide abundant sunshine that favors agricultural production (Hao et al., 2023; Gao et al., 2024; Tian et al., 2025).

Wheat, the most important cereal crop in Xinjiang, is cultivated in geographically isolated production areas separated from other Chinese wheat regions by deserts and mountains. Xinjiang shares 5,600 km of borderlines with eight countries (Russia, Kazakhstan, Kyrgyzstan, Tajikistan, Pakistan, Mongolia, India). As one of China’s primary wheat production zones, Xinjiang maintains 600,000–750,000 hectares of wheat cultivation area with an annual output of approximately 3.75 million tons (Wan et al., 2015). Wheat is widely distributed across all agricultural regions except Turpan (Zhang et al., 2024), with irrigated wheat dominating most areas and rainfed wheat limited to minor zones.

### 2.2 Sampling and measurement

#### 2.2.1 Wheat sampling and harvest index calculation

During the wheat maturity period from 2022 to 2023, sampling was conducted in 11 typical wheat-growing counties/cities (3 in 2022 and 8 in 2023) across eight prefectures in Xinjiang (**Figure 1**). In each county/city, five representative wheat-growing villages were selected, and two typical wheat fields were chosen per village. Within each sampling plot, wheat was manually cut close to the ground from a 5 m² area using the “five-point sampling method.” Surface dust and other weight-affecting impurities were removed before the samples were packed and transported to the laboratory for harvest index determination.

All seeds were manually separated from the wheat plants and dried in an oven at 105°C for 30 minutes. The oven temperature was then adjusted to 75°C until a constant weight was achieved. The wheat yield and aboveground biomass were weighed and recorded separately. The wheat harvest index (HI) was calculated as follows (Bilandžija et al., 2023; Du et al., 2024):


Harvest Index(HI)=Yield/Aboveground Biomass(AGB)

where “Yield” refers to wheat yield (kg/m²), and “AGB” represents aboveground biomass (kg/m²).

#### 2.2.2 Geographic-climatic factors

Geographical coordinates (longitude(lon), latitude(lat) and altitude (alt)) of sampling sites were recorded *in situ* using Huace X90-series GPS receivers during wheat sampling. For soil accumulated temperature (SAT), we calculated the total effective accumulated soil temperature during the wheat growing season (March to July) using daily temperatures ≥ 10°C. All temperature data were obtained from the National Meteorological Science Data Center (http://data.cma.cn/).

#### 2.2.3 Agronomic management factors

Plant height (H): At wheat harvest, more than five plots were selected in each sampling area, with over 10 wheat plants randomly measured per plot using a 1 mm-precision steel tape. The height from the plant base to the apical growing point was recorded, and the average across all plots represented the plant height for that sampling area. Wheat yield (yield): The dry weight of wheat grains from each 5 m² sampling plot was measured to calculate yield per unit area. Aboveground biomass (AGB): The harvested 5 m² wheat samples were oven-dried to constant weight to determine AGB per unit area. Irrigation (irrigation): Based on farmers’ cultivation practices, seasonal water availability, and the arid climate, wheat irrigation regimes were classified as either irrigated or rainfed.

#### 2.2.4 Soil nutrient factors

Soil samples (0–20 cm depth) were collected from each plot using a soil auger following the “five-point sampling method”. After homogenization, air-drying under natural conditions, and removal of visible debris, samples were ground for analysis. The measurement protocols for soil nutrients were as follows: Soil Organic Matter (SOM, g/kg): Potassium dichromate oxidation-external heating method; Total Nitrogen (TN, g/kg): Sulfuric acid-perchloric acid digestion (Foss Kjeltec 1035 auto-analyzer); Total Phosphorus (TP, g/kg): Acid digestion-molybdenum antimony colorimetry (Agilent Cary 60 UV-Vis spectrophotometer); Total Potassium (TK, g/kg): Acid digestion-atomic absorption spectrometry (Thermo Scientific S-series AAS); Alkali-hydrolyzable Nitrogen (ASN, mg/kg): Alkali diffusion method; Available Phosphorus (AP, mg/kg): Sodium bicarbonate extraction-molybdenum antimony colorimetry (Agilent Cary 60 UV-Vis spectrophotometer); Available Potassium (AK, mg/kg): Ammonium acetate extraction-atomic absorption spectrometry (Thermo Scientific S-series AAS); pH: Measured using a pH meter (PHS-2F); Soil Electrical Conductivity (EC, mS/cm): Determined with a conductivity meter (DDSJ-308F); Soil Water Content (SWC, g/cm³): Measured by oven-drying core samples (Φ200) at 105°C to constant weight; Bulk Density (BD, g/cm³): Calculated from the dry weight of core samples (Φ200) per unit volume.

### 2.3 Data analysis

Spatial interpolation was conducted through Kriging analysis in ArcGIS 10.8. As a geostatistical method accounting for spatial uncertainty, Kriging incorporates sample spatial relationships via variogram modeling and structural analysis to generate optimal unbiased estimates for unsampled locations (Matheron, 1963; Shen et al., 2023). The semivariogram, the core algorithm component, quantifies spatial variability as a function of inter-sample distances, enabling best linear unbiased predictions (BLUPs) with uncertainty estimates. We selected ordinary Kriging after comparative testing due to its superior model accuracy and economic decision reliability versus simple Kriging (Daya and Bejari, 2015), implementing it with second-order polynomial trend functions. Prior to interpolation, Shapiro-Wilk normality tests (R 4.4.2) confirmed data normality (*p* > 0.05). Optimal modeling of HI-geographic coordinate (longitude/latitude) relationships was achieved using GS+ 9.0. Interpolation accuracy was assessed by two metrics: mean error (ME, Equation 1, optimal at 0), and root mean square error (RMSE, Equation 2, smaller values indicate greater reliability) (Wieskotten et al., 2024).


(1) $$ME=\frac{\sum_{i=1}^{N}[Z(s_{i})-z(s_{i})]}{n}$$

(2) $$RMSE=\sqrt{\frac{\sum_{i=1}^{N}{\left[Z(s_{i})-z(s_{i})\right]}^{2}}{n}}$$

Statistical analyses were conducted using R 4.4.2. First, intra-group correlation analysis between wheat harvest index (HI) and its influencing factors was performed using the “psych” package, with visualization of correlation differences across irrigation regimes. Second, the “randomForest” package was employed to build random forest models for predicting HI-driving factors, followed by variable importance ranking. Third, partial least squares path modeling (PLS-PM) was implemented via the “plspm” package to systematically assess the integrated effects of multidimensional factors (geographic-climatic, agronomic, and soil nutrient variables) on HI. Finally, linear regression analyses were conducted using the “ggpmisc” package to quantify relationships between key drivers and HI.

## 3 Results

### 3.1 Spatial variation of wheat harvest index and irrigation effects in arid regions

**Figure 2:**
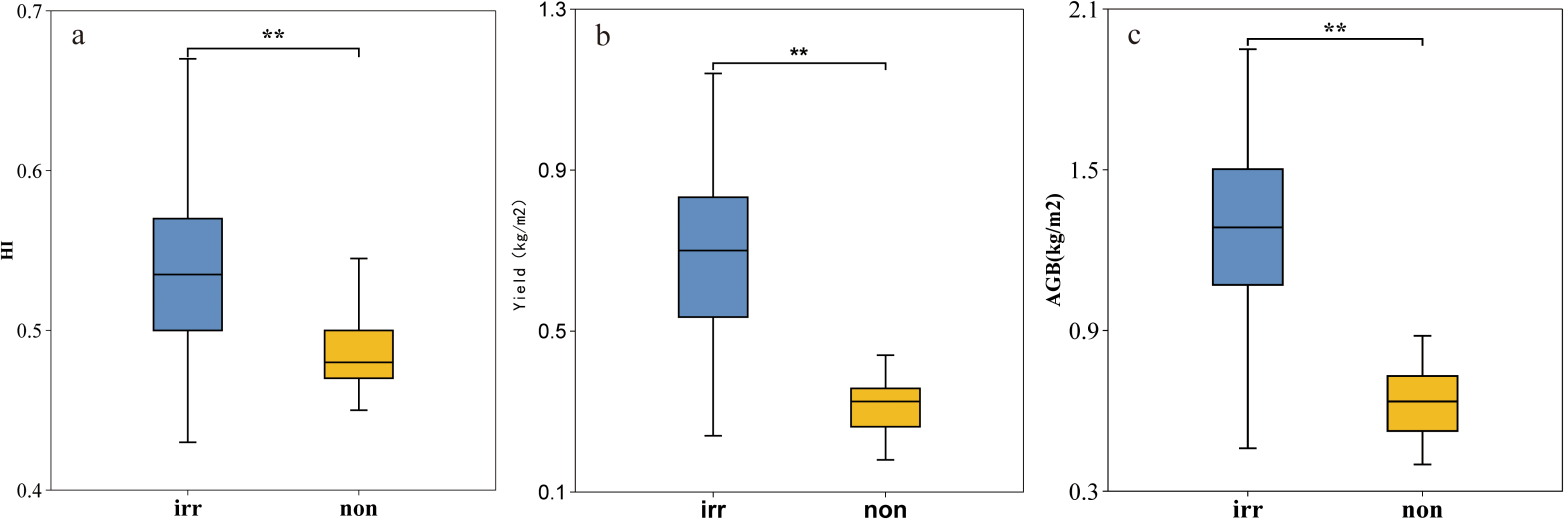
Irrigation effects on wheat productivity. **(a–c)**, HI **(a)**, yield **(b)**, and AGB **(c)** comparisons between irrigated (irr, blue) and rainfed (non, orange) plots (**p<0.01).

The study revealed significant spatial variability in wheat harvest index (HI) across arid regions, with values ranging from 0.43 to 0.67 (mean ± SD: 0.52 ± 0.03). Irrigation significantly enhanced HI levels (*p* < 0.01), demonstrating a 10.2% increase in irrigated(irr) wheat HI (0.54 ± 0.05) compared to rainfed systems(non) (0.49 ± 0.02). Significant differences were observed among irrigation regimes in their effects on wheat productivity (**Figure 2**). Irrigated wheat showed superior performance over rainfed cultivation (*p* < 0.01) across all measured parameters: grain yield (+23.5%), aboveground biomass (AGB, +18.7%), and harvest index (HI, +10.2%).

**Figure 3:**
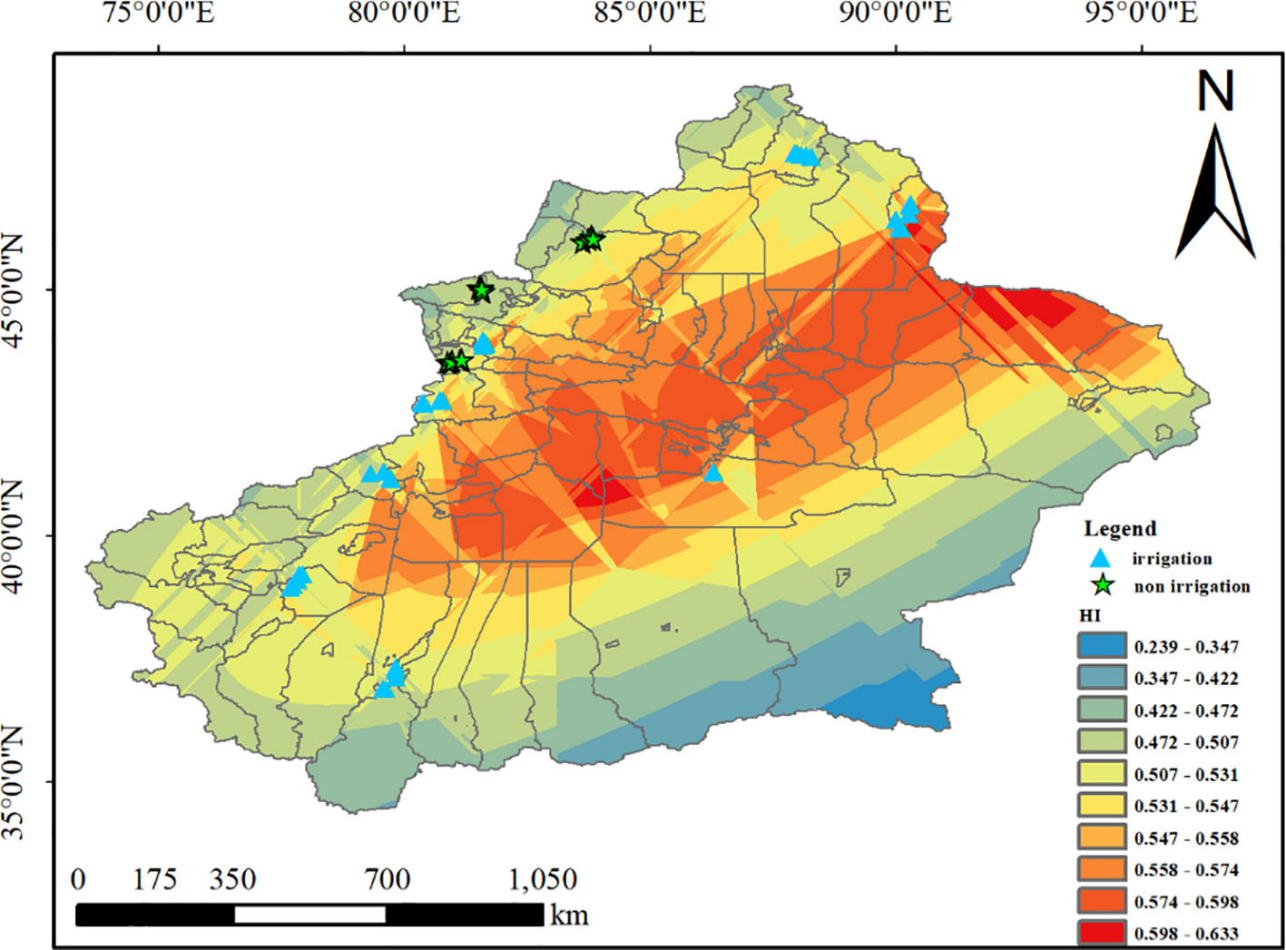
Spatial patterns of wheat HI in arid regions.

Spatial analysis using ordinary Kriging interpolation (**Figure 3**) demonstrated significant spatial heterogeneity in wheat harvest index (HI) across arid regions, characterized by the following patterns: HI values predominantly ranged between 0.4-0.6, exhibiting a distinct spatial gradient with higher values in northern areas, lower values in southern regions, and depressed values in central zones relative to peripheral areas. Key geographical patterns were observed: Irrigation-dependent cultivation: Most wheat fields in Xinjiang employed irrigated systems, showing a consistent north-high-south-low HI distribution (**Supplementary Figure 1a**). Rainfed systems: Mountainous border areas relying on natural precipitation developed unique rainfed cultivation, maintaining similar north-south HI gradients (**Supplementary Figure 1b**).

Spatial variability of wheat harvest index (HI) was characterized through semivariogram analysis. The nugget ratio (C_0_/(C_0_+C)), representing the proportion of random variation to total variability, indicated: strong spatial dependence when <25%, moderate at 25-75%, and weak when >75%. The wheat harvest index (HI) in arid regions exhibited a nugget-to-sill ratio of 11.97%, indicating strong spatial autocorrelation. Combined with coefficient of variation (CV) analysis, the overall wheat HI in Xinjiang (a representative arid zone) was 4.3% (**Table 1**), demonstrating low data variability and minimal fluctuations in raw values. The root mean square error (RMSE) approached zero, confirming high interpolation accuracy and reliability of the results (**Table 1**). Both irrigated and rainfed wheat HI systems displayed moderate spatial autocorrelation, low data variability, and robust model precision (see **Supplementary Table 1**; **Table 2**).

**Table 1:** Parameters of semivariogram models for wheat harvest index in arid regions.

Objective	Nuggget C_0_	Sill C+C_0_	RANGE A_0_(Km)	Proportion (C_0_/C+C_0_)	Theoretical model	CV	ME	RMSE
Harvest index	0.00013	0.00095	0.2252	11.97%	Gaussian	4.3%	0.0629	0.1218

Gaussian, Gaussian model; CV, Combined with coefficient of variation.

**Table 2:** Significant effects of different types of factors on wheat harvest index in arid regions (**p < 0.01, *p < 0.05. red = positive correlation, green = negative correlation).

Factor type	Factors	*P* value		
		all	irr	non
Geographic-climatic factors	irrigation	0.001**	–	–
	lon	0.004**	0.011*	0.178
	lat	0.614	0.019*	0.865
	alt	0.347	0.143	0.123
	SAT	-0.040*	0.058	0.118
Agronomic management factors	yield	0.001**	0.001**	0.087
	H	-0.001**	-0.042*	0.002**
	AGB	0.004**	0.200	0.540
Soil nutrient factors	pH	-0.012*	-0.046*	0.412
	EC	0.360	-0.038*	0.427
	SWC	0.001**	0.442	-0.001**
	BD	0.066	0.264	0.001**
	SOM	0.068	0.638	0.385
	TN	0.027*	0.515	0.001**
	TP	0.009**	0.403	0.643
	TK	0.990	0.120	0.002**
	ASN	0.031*	0.561	0.003**
	AP	0.004**	0.524	0.002**
	AK	0.172	0.333	0.074

### 3.2 Key driving factors of wheat harvest index in arid regions

#### 3.2.1 Correlation analysis between wheat harvest index and influencing factors

**Figure 4:**
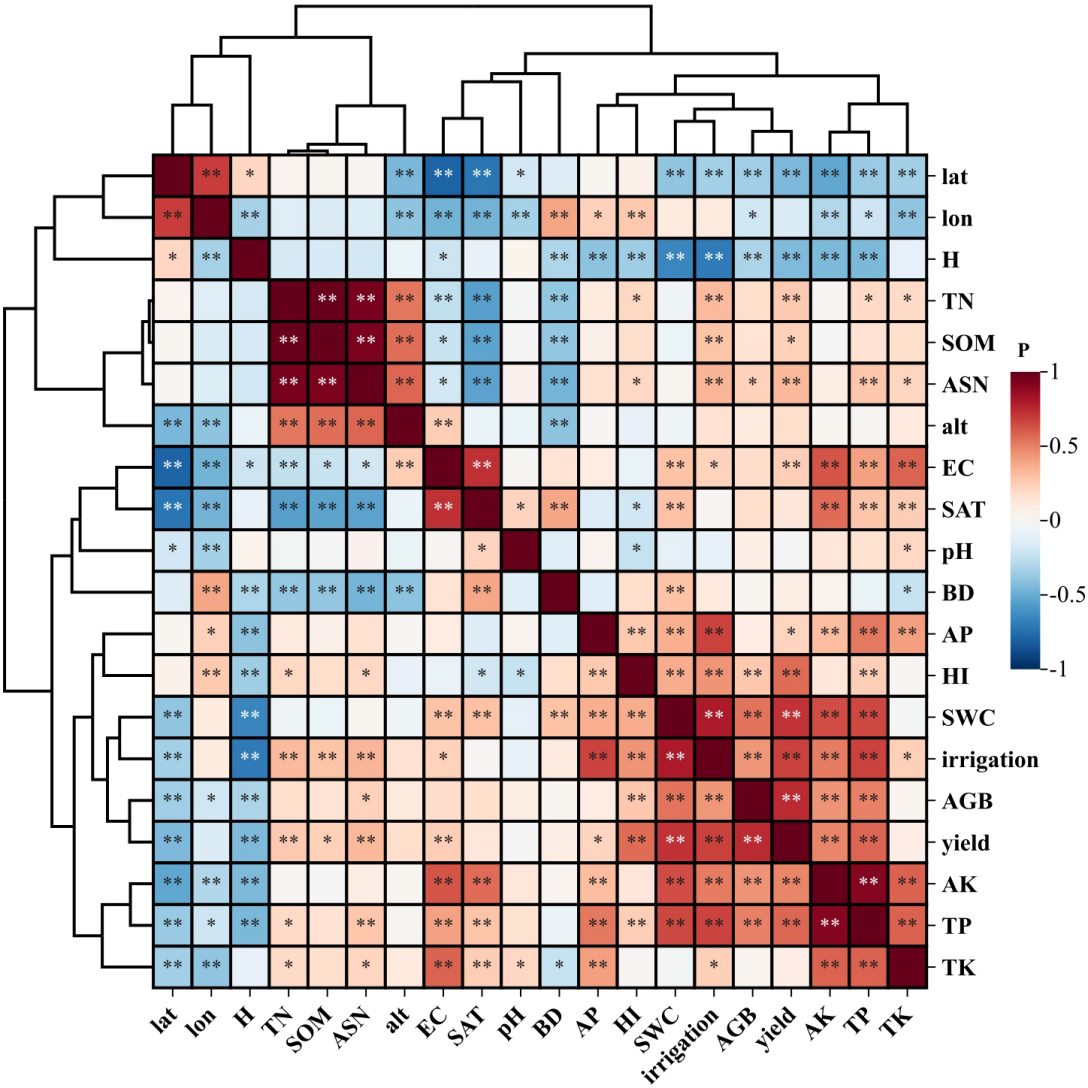
Correlation heatmap of wheat HI-associated factors in arid regions (**p < 0.01, *p < 0.05).

Based on Pearson correlation analysis and hierarchical clustering results (**Figure 4**), the association characteristics between wheat harvest index (HI) and environmental factors in arid regions were revealed: Agronomic management factors: HI showed significant positive correlations with yield, aboveground biomass (AGB), and irrigation (*p* < 0.01), but a significant negative correlation with plant height (H) (*p* < 0.01). Soil nutrient factors: HI exhibited significant positive correlations with total phosphorus (TP), available phosphorus (AP), and soil water content (SWC) (*p* < 0.01). Geographic-climatic factors: Only longitude (lon) demonstrated a significant negative correlation with HI (*p* < 0.01). The hierarchical clustering dendrogram (**Figure 4**) further indicated that: AP clustered most closely with HI, suggesting that available phosphorus is the most critical driver of HI. HI, AP, SWC, irrigation, AGB, and yield formed a core group of key driving factors, highlighting their synergistic effects on HI. Geographic-climatic factors formed a distinct cluster, reflecting their indirect influence on HI through interactions with other variables.

**Figure 5:**
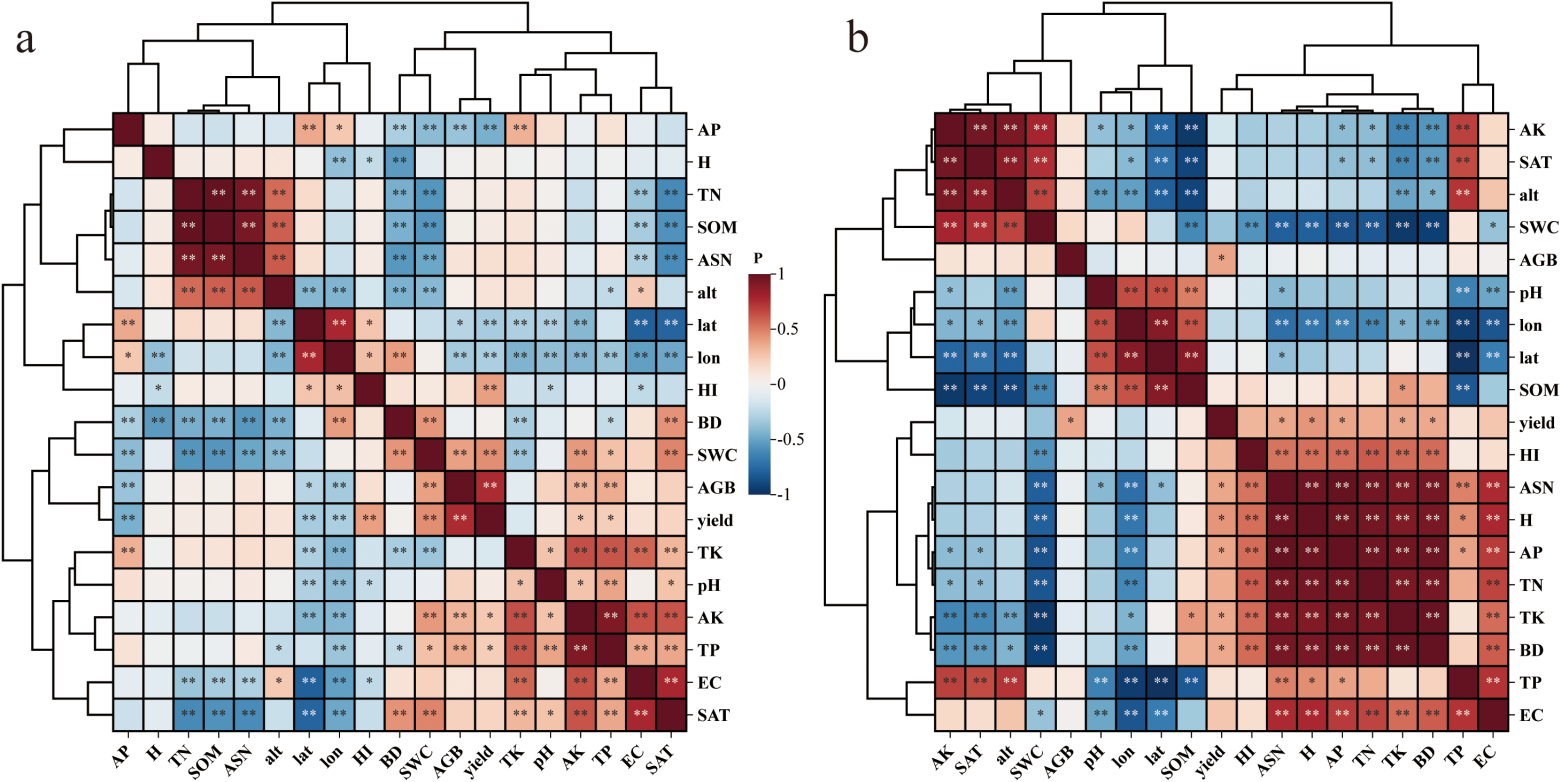
Correlation heatmap of wheat HI drivers under different water regimes in arid regions (**p < 0.01, *p < 0.05). **(a)** Irrigated wheat; **(b)** Rainfed wheat. Color gradient: red = positive, blue = negative correlations.

For irrigated wheat, the harvest index (HI) showed a significant positive correlation (*p* < 0.01) only with yield among the agronomic management factors (**Figure 5a**). In contrast, for rainfed wheat, HI exhibited: A significant positive correlation (*p* < 0.01) with plant height (H) among agronomic factors (**Figure 5b**). Significant positive correlations (*p* < 0.01) with bulk density (BD), total potassium (TK), total nitrogen (TN), available phosphorus (AP), and available soil nitrogen (ASN) among soil nutrient factors. A significant negative correlation (*p* < 0.01) with soil water content (SWC). The hierarchical clustering dendrogram further revealed distinct patterns: Irrigated wheat HI did not cluster closely with other factors but instead formed a major group with longitude (lon) and latitude (lat), indicating that geographical location is a key driver of HI in irrigated wheat systems. Rainfed wheat HI clustered most closely with AP, forming a distinct subgroup that further grouped with ASN, H, TN, TK, and BD, collectively representing the dominant influencing factors for rainfed wheat HI. The effects of different factor types on the overall, irrigated, and rainfed wheat Harvest Index (HI) exhibited significant differences in their statistical significance (**Table 2**).

#### 3.2.2 Importance analysis of influencing factors based on random forest model

**Figure 6:**
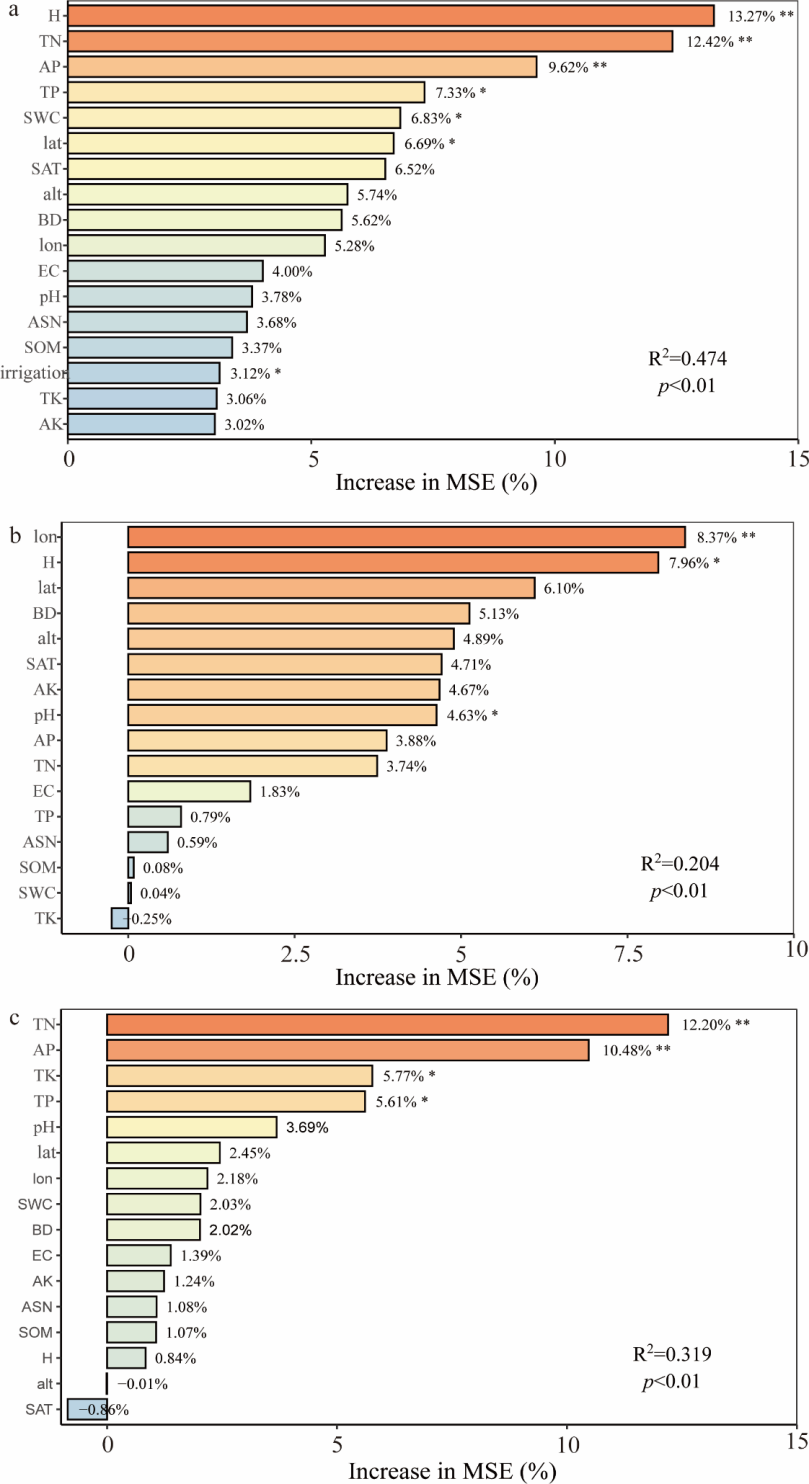
Importance ranking of influencing factors in random forest models(**p < 0.01, *p < 0.05. **(a)** Random forest prediction of factors affecting overall wheat HI in arid regions. **(b)** Random forest prediction for irrigated wheat HI. **(c)** Random forest prediction for rainfed wheat HI).

A Random Forest model was employed to analyze the relative importance and contribution values of various factors affecting wheat Harvest Index (HI) in arid regions, aiming to identify critical influencing factors. Given that HI is directly calculated from yield and aboveground biomass (AGB), these two variables were excluded from the Random Forest analysis to avoid circular reasoning. The results demonstrated that the model explained 47.4% of the variance in overall wheat HI across arid regions (**Figure 6a**). Plant height (H, 13.27%) and total nitrogen (TN, 12.42%) emerged as key drivers (importance >10%), followed by available phosphorus (AP, 9.62%), total phosphorus (TP, 7.33%), soil water content (SWC, 6.83%), latitude (lat, 6.69%), soil available potassium (SAT, 6.52%), altitude (alt, 5.74%), bulk density (BD, 5.62%), and longitude (lon, 5.28%), classified as secondary factors (importance 5-10%).

For irrigated wheat systems (**Figure 6b**), the hierarchy of factor importance was: longitude (lon, 8.37%), plant height (H, 7.96%), latitude (lat, 6.10%), bulk density (BD, 5.13%) (importance >5%). In contrast, rainfed wheat systems (**Figure 6c**) exhibited distinct prioritization: total nitrogen (TN, 12.20%), available phosphorus (AP, 10.48%), total potassium (TK, 5.77%), total phosphorus (TP, 5.61%) (importance >5%). While soil nutrient factors collectively dominated in importance across all systems, the relative contributions of different factor types (geographic, climatic, and management) to HI variation diverged markedly among overall, irrigated, and rainfed wheat systems (**Table 3**).

**Table 3:** Differences in the relative importance of factor types on wheat Harvest Index (HI) in arid regions (**p < 0.01, *p < 0.05).

Factor type	Factors	Important value(%)		
		all	irr	non
Geographic-climatic factors	lon	5.28	8.37**	2.18
	lat	6.69*	6.1	2.45
	alt	5.74	4.89	-0.01
	SAT	6.52	4.71	-0.86
Agronomic management factors	H	13.27**	7.96*	0.84
	irrigation	3.12*	–	–
Soil nutrient factors	TN	12.42**	3.74	12.20**
	AP	9.62**	3.88	10.48**
	TP	7.33*	0.79	5.61*
	SWC	6.83*	0.04	2.03
	BD	5.62	5.13	2.02
	EC	4.00	1.83	1.39
	pH	3.78	4.63*	3.69
	ASN	3.68	0.59	1.08
	SOM	3.37	0.08	1.07
	TK	3.06	-0.25	5.77*
	AK	3.02	4.67	1.24
R^2^		R^2^ = 0.474	R^2^ = 0.204	R^2^ = 0.319
*p*		*p*<0.01	*p*<0.01	*p*<0.01

#### 3.2.3 Analysis of factors affecting wheat harvest index using structural equation modeling

**Figure 7:**
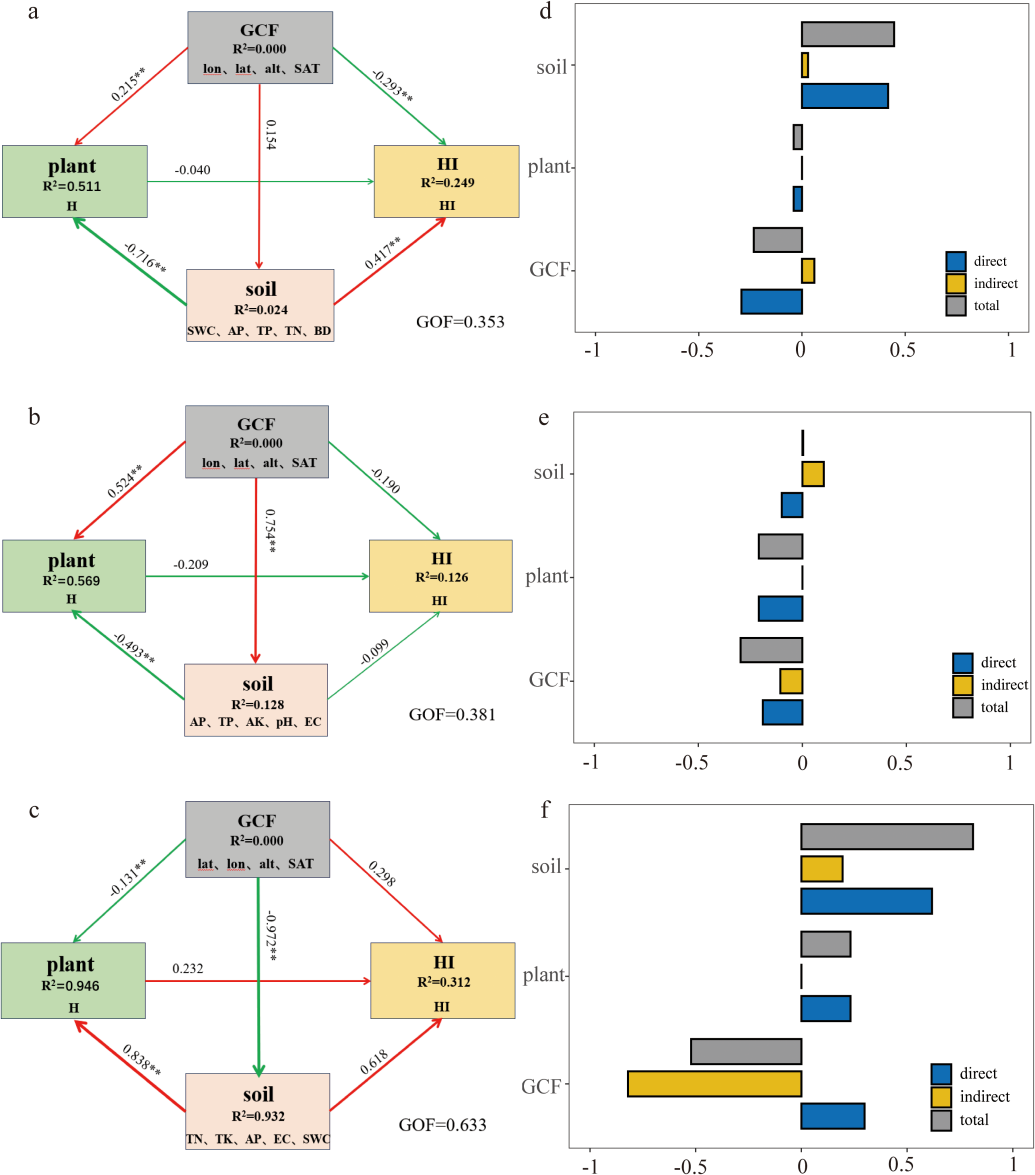
Interactions among factor categories in the PLS-PM structural equation model(**p < 0.01, *p < 0.05. Left panels: Effects and intensity of three factor categories on wheat HI for **(a)** overall arid regions, **(b)** irrigated, and **(c)** rainfed systems. Right panels: Bar plots showing **(d)** overall, **(e)** irrigated, and **(f)** rainfed wheat HI factor effects derived from SEM, including direct, indirect, and total effect magnitudes. Numeric labels on arrows indicate standardized path coefficients. Arrow thickness reflects coefficient magnitude. Color coding: red = positive correlation, blue = negative correlation. Factor importance: Leftward position within each factor category indicates stronger HI influence).

Based on the key variables identified through prior analyses, we employed Structural Equation Modeling (SEM) to investigate the interactions among the three factor types (geographic-climatic, soil nutrient, and agricultural management) and quantify their direct and indirect contributions to the Harvest Index (HI). This approach enables systematic visualization of causal pathways, effectively disentangling the complex interdependencies between these factors and HI through standardized path coefficients and model fit indices. We selected: All factors from Geographic-climatic factors (GCF). Plant height (H) as the representative of wheat plant characteristics (plant). The top five most important factors from soil nutrient factors (soil). We then constructed structural equation models (SEM) for: The overall wheat samples, Irrigated wheat systems and Rainfed wheat systems (**Figure 7**).

Key findings from path analysis: Overall wheat HI (**Figure 7a**): Soil nutrients showed the strongest positive effect. GCF exhibited significant negative effects. Plant characteristics had weak negative effects. Irrigated wheat (**Figure 7b**): All three factor categories negatively affected HI. Effect strength: Plant > GCF > Soil. Rainfed wheat (**Figure 7c**) (most stable model, GOF=0.633): All three factor categories positively influenced HI, Effect strength: Soil > GCF > Plant.

#### 3.2.4 Linear regression analyses of the influencing factors of wheat harvest index in arid regions

**Figure 8:**
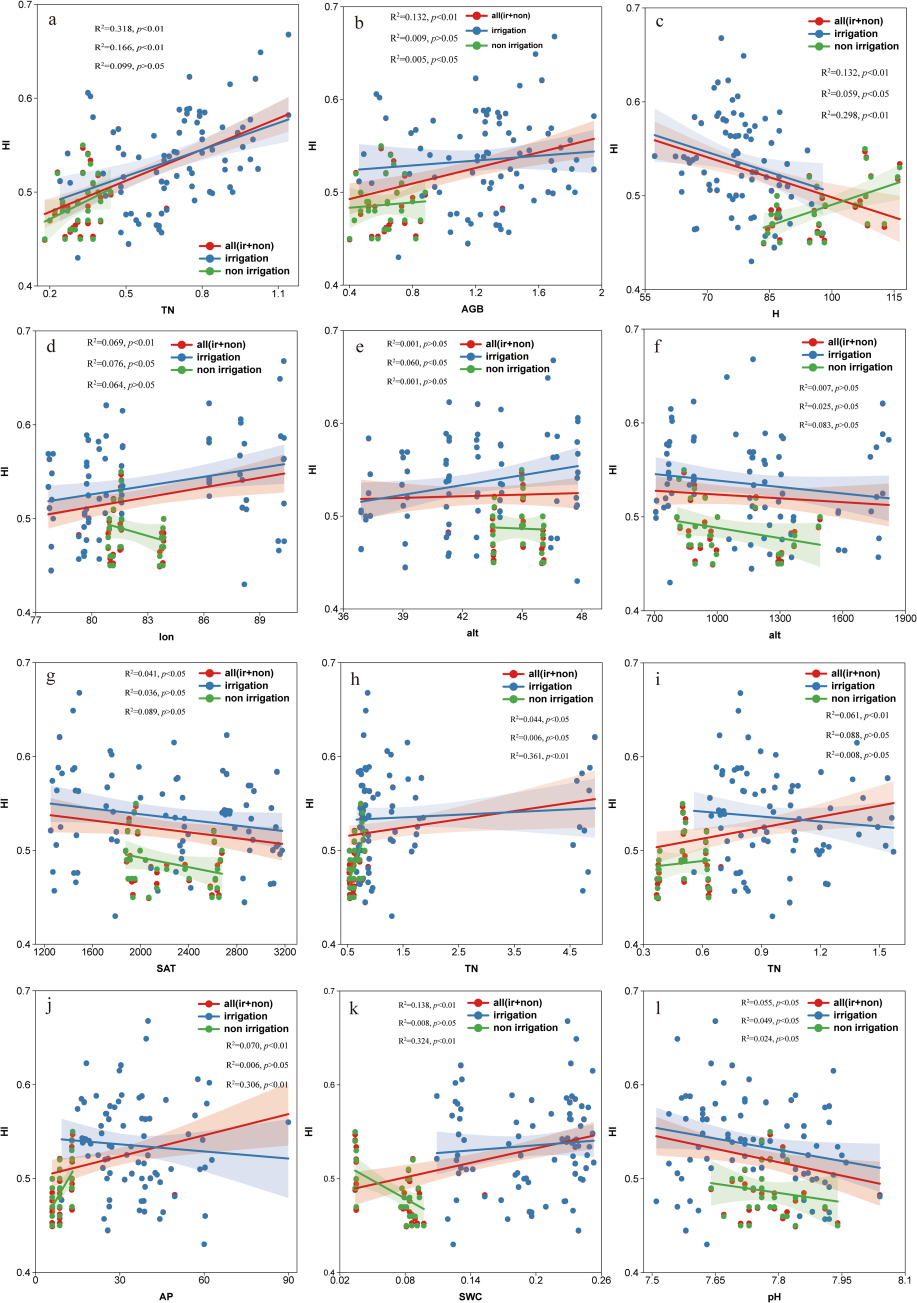
Linear regression analysis between influencing factors and wheat harvest index HI in arid regions. (Subfigure panels: **(a)** yield(kg/m^2^) vs. HI. **(b)** Aboveground biomass (AGB, kg/m^2^) vs. HI. **(c)** Plant height (H, m) vs. HI. **(d)** longitude(°) vs. HI. **(e)** latitude(°) vs. HI. **(f)** altitude vs. HI. **(g)** Soil accumulated temperature (SAT, °C) vs. HI. **(h)** Total nitrogen (TN, g/kg) vs. HI. **(i)** Total phosphorus (TP, g/kg) vs. HI. **(j)** Available phosphorus(AP, mg/kg) vs. HI. **(k)** Soil water content (SWC, g/cm^3^) vs. HI. **(l)** Soil pH vs. HI).

Based on the results of correlation analysis, Random Forest and Structural Equation Modeling(SEM), a linear fit of the three types of significant influences on the impact factors to wheat HI systematically quantifies how changes in these key drivers modulate wheat HI trends in arid regions. The similarities and differences between irrigated and rainfed wheat HI trends can also be visualized. It was found that Geographic-climatic factors had relatively less effect on wheat HI. Overall, wheat HI increased with increasing longitude (lon) and latitude (lat), and in irrigated wheat, HI increased with increasing latitude and longitude; whereas in rainfed wheat, HI had the opposite trend, decreasing with increasing latitude and longitude (**Figures 8d, e**). Meanwhile, wheat HI showed a decreasing trend with increasing altitude (alt) and soil accumulated temperature (SAT) (**Figures 8f, g**).

Agronomic management factors significantly influenced wheat HI, with plant biomass (AGB) and yield (yield) having the most direct effect on wheat HI, which significantly increased as they increased. In addition, AGB, yield and HI were significantly higher in irrigated wheat than in rainfed wheat (**Figures 8a, b**). Overall, there was a negative correlation between HI and water stress (H), with irrigated wheat showing a similar trend, whereas rainfed wheat showed an increase in HI when water stress increased instead (**Figure 8c**).

Soil nutrient factors are particularly important in influencing HI of wheat in arid regions, which is the basic security for wheat growth. The present study showed that the total nitrogen (TN) content of irrigated wheat was significantly higher than that of rainfed wheat, and HI increased with increasing TN, with rainfed wheat showing a more pronounced increase in this respect (**Figure 8h**). Overall, wheat HI was elevated with the increase in effective phosphorus (AP); in irrigated wheat, although HI showed a slight decrease with the increase in AP, the AP content was significantly higher in irrigated wheat than in rainfed wheat. The effect of AP on HI was more sensitive in the rainfed case, where HI was significantly elevated (**Figure 8i**). In addition, soil water content (SWC) was significantly higher in irrigated wheat than in rainfed wheat, and HI generally increased with increasing SWC, where HI was positively correlated with SWC in irrigated wheat, whereas HI decreased sharply with increasing SWC in rainfed wheat (**Figure 8j**). Finally, HI also increased with increasing soil density (BD), but rainfed wheat responded more sensitively to this (**Figure 8k**). It is worth noting that in arid regions, where the pH of wheat soils is alkaline, HI decreased overall with increasing pH (**Figure 8l**).

## 4 Discussion

### 4.1 Spatial distribution characteristics of wheat harvest index in arid regions

The wheat harvest index (HI) in arid regions exhibits a distinct spatial pattern of “high in the center, low at the edges, and higher in the north than in the south”, with notable differences in HI distribution under different irrigation methods. This distribution pattern is closely related to regional topographic features and agricultural management conditions. As a typical representative of arid regions in northwestern China, Xinjiang’s border areas feature higher altitudes and rugged terrain, which hinder mechanized farming and the implementation of management practices, leading to significantly lower harvest indices in these peripheral regions compared to the central irrigated agricultural areas (**Figure 2**). This finding aligns with the spatial distribution patterns of agricultural productivity in global arid regions. Additionally, precipitation in Xinjiang is concentrated mainly in summer, with spatial distribution showing higher rainfall in northern Xinjiang than in southern Xinjiang (Jiang et al., 2013; Zeming et al., 2018; Hu et al., 2021; AiHaiti et al., 2023), indirectly contributing to the north-high, south-low distribution pattern of wheat HI in arid regions.

From an international comparative perspective, there are significant differences in the wheat harvest index (HI) across different ecological regions. In the temperate regions of Northwestern Europe, the theoretical HI of winter wheat can reach 0.62 (Austin et al., 1980), while observed values generally remain around 0.50 (Wang et al., 2020). In contrast, under irrigated conditions, spring wheat HI is typically below 0.50 (Pradhan et al., 2019). In Australia, under rainfed conditions, wheat HI ranges only from 0.24 to 0.45. These variations primarily stem from reduced assimilate translocation efficiency due to post-anthesis water stress. Notably, the HI of rainfed wheat in China’s Loess Plateau ranges from 0.28 to 0.56 (Hu et al., 2019), yet there remains a significant gap compared to the theoretical maximum of 0.62 for modern winter wheat (Wang et al., 2020). Some studies (Foulkes et al., 2011) suggest that wheat HI can reach up to 65%, but it has largely remained unchanged for decades.

This study reveals that the harvest index (HI) of irrigated wheat in arid regions reaches 0.43-0.67, with some experimental sites approaching the recognized theoretical maximum, potentially attributable to the excessive allocation ratio to economic organs under intensive irrigation management. In contrast, rainfed wheat systems maintain an HI of 0.45-0.55, consistent with global observations in arid regions. These findings suggest that: 1) under irrigated conditions, the potential for yield improvement through HI enhancement is approaching biological limits; 2) although rainfed systems possess room for improvement (falling 5–15 percentage points short of the theoretical maximum), progress is constrained by water stress and requires breakthroughs through optimizing water use efficiency or alternative approaches. This understanding provides crucial theoretical guidance for developing wheat production strategies in arid regions: irrigated areas should shift focus toward synergistic yield-quality improvement, while rainfed systems should continue targeting HI enhancement as a core breeding objective.

### 4.2 Key driving factors of wheat harvest index in arid regions

In arid regions, the harvest index (HI) of wheat is co-regulated by Geographic-climatic factors, agronomic management factors, and soil nutrient factors, though their relative contributions varied significantly. The study revealed that soil nutrient factors exerted the most pronounced influence, followed by agronomic management factors, while the impact of Geographic-climatic factors was relatively weaker. Additionally, the relative strength of these driving factors differed across irrigation regimes.

#### 4.2.1 Impact of geographic-climatic factors on wheat harvest index in arid regions

Geographic-climatic factors exhibit relatively limited but discernible spatial influence on wheat HI in arid regions. The study reveals that HI increases with longitude but decreases with accumulated effective soil temperature. In Xinjiang, a typical arid region, the distinct winter-cold and summer-hot climate renders water availability the primary limiting factor for wheat cultivation. Water supply patterns play a decisive role in determining HI (van den Boogaard et al., 1996). Compared to irrigated conditions, wheat grown under natural precipitation shows reduced yield, biomass, and heading days, along with significantly lower HI. Notably, drought-resistant cultivars typically demonstrate higher water-use efficiency than irrigated varieties (van den Boogaard et al., 1996), indicating differential adaptation strategies to water stress among wheat ecotypes. Globally, water deficit remains a key ecological constraint limiting crop productivity (Zhang et al., 2006). However, arid agricultural systems face dual challenges of water scarcity and inefficient utilization, severely constraining sustainable crop production (Ogola et al., 2002). Consequently, optimized water management remains crucial for improving wheat HI in arid regions.

#### 4.2.2 Effects of agronomic management factors on wheat harvest index in arid regions

The harvest index (HI) has been demonstrated to be a modifiable factor in crop production. The extent to which agricultural systems are influenced by climatic conditions varies depending on crop species, region, and management practices (Schlindwein et al., 2015). Our findings similarly indicate that agronomic management factors significantly affect wheat HI in arid regions, where wheat HI increases with higher grain yield and aboveground biomass (AGB), but decreases with plant height. Given the inherent upper limit of crop HI, recent introductions of dwarfing genes have reduced wheat plant height, altering dry matter partitioning while improving lodging resistance and yield potential, thereby facilitating HI enhancement (Brancourt-Hulmel et al., 2003; Zhang et al., 2012; Ukozehasi et al., 2022; Xi et al., 2024). Variations in crop HI are primarily attributable to differences in crop management. Research shows that wheat HI exhibits a negative correlation with the stem-to-AGB ratio but a positive correlation with the spike-to-AGB ratio. Foulkes et al. (2011) estimated that a 10% increase in AGB could theoretically raise HI to 0.64. Under drought stress conditions, the positive effect of HI on yield becomes more directly pronounced (Yang et al., 2000; Peltonen-Sainio et al., 2008; Zhang et al., 2012).

Significant differences in wheat harvest index (HI) were observed among different irrigation methods. Irrigated wheat demonstrated significantly higher grain yield, aboveground biomass (AGB), and HI compared to rainfed wheat (*p* < 0.01). These findings are consistent with a meta-analysis by Yang et al. (2022), which reported that irrigation can increase wheat yield by an average of 50%. Irrigation directly affects grain yield and HI (Ma et al., 2025) by regulating soil moisture conditions during grain filling (Uddling et al., 2008). In rainfed systems, natural precipitation serves as the sole water source, determining soil water availability (Noor et al., 2023). Under limited irrigation conditions, yield reduction caused by water constraints depends on both the severity and duration of soil water deficit. The impact of water deficit on crop yield varies according to specific phenological stages, with the most sensitive stages showing regional variations (Mishra et al., 1991). Since these variations are associated with regional differences in environmental conditions and agronomic practices, location-specific information is required to develop and optimize limited irrigation strategies. Optimal irrigation levels, nitrogen application rates, and cultivar selection can significantly improve winter wheat cultivation area, AGB, and yield, while nitrogen uptake increases with both irrigation and nitrogen inputs (Bingjun et al., 2018; Li et al., 2021). A key question regarding wheat (*Triticum aestivum L*.) HI in arid regions is whether HI manipulation in crop production can simultaneously achieve the dual objectives of increasing grain production and improving water conservation (Yang et al., 2000; Yang and Zhang, 2010).

#### 4.2.3 Effects of soil nutrients factors on wheat harvest index in arid regions

Soil nutrient factors exert significant influences on wheat harvest index (HI) in arid regions, including soil nitrogen (N), phosphorus (P), water content, and pH. In long-term experiments, these factors induce changes in soil physical and chemical properties through differential inputs of mineral nutrients and organic matter, thereby directly affecting crop yield (Girma et al., 2007; Wang et al., 2017). Phosphorus represents one of the most critical limiting factors for plant growth (Albayrak et al., 2009). In arid soils, phosphorus deficiency inhibits wheat root development, consequently affecting growth rates (Zhao et al., 2024). Our results demonstrate significant positive correlations between soil total phosphorus (TP), available phosphorus (AP) and wheat HI in arid regions (*p* < 0.01). Soil available phosphorus not only serves as a key driver in soil carbon, nitrogen, phosphorus and sulfur cycles (Zhao et al., 2024), but also constitutes an essential nutritional source determining wheat harvest index.

Furthermore, both soil total nitrogen and available nitrogen demonstrated significant effects on wheat harvest index (HI) in arid regions (p < 0.05). Hay (1995) reported that nitrogen fertilizer had minimal impact on HI when applied at optimal rates. However, studies have indicated that in water-limited environments, excessive nitrogen application can lead to reduced yield and HI due to over-stimulated vegetative growth, which is associated with decreased post-anthesis carbon assimilation caused by soil water deficit (Herwaarden et al., 1998; Porker et al., 2020). Divergent perspectives exist regarding the effects of nitrogen application strategies on HI, likely attributable to the complex interactions between environmental factors and plant growth/yield formation. The prevailing view suggests that excessive nitrogen application may lead to decreased HI (Porker et al., 2020). Research has also revealed a positive correlation between wheat grain protein content and soil nitrogen availability, but an inverse relationship with actual grain yield (Prystupa et al., 2018). The ratio of soil available nitrogen to grain yield shows close association with grain protein content and has been proposed as a useful indicator for estimating the required amount of available nitrogen to achieve target yields (Prystupa et al., 2018). Given the generally positive correlation between soil available nitrogen and crop nitrogen uptake, it can be inferred that a similar relationship exists between grain nitrogen concentration and nitrogen uptake per unit grain yield in wheat (Gómez et al., 2021). Some studies have observed greater nitrogen uptake under irrigated conditions compared to rainfed systems (Zhang et al., 2017; Yan et al., 2022). Nevertheless, the underlying mechanisms through which soil nitrogen influences wheat HI in arid regions require further investigation.

A significant positive correlation was observed between soil water content and wheat harvest index (HI) in arid regions (p < 0.01), with HI progressively increasing as soil moisture levels rose. Given that wheat primarily grows under rainfed conditions in these areas, its growth and yield are highly dependent on both water availability and fertilizer inputs. Consequently, investigating the relationships among soil moisture, fertilizer levels, and crop growth is crucial for developing more effective dryland management practices (Halitligil et al., 2000; Huang et al., 2003). The synergistic interaction between soil water and fertilizers plays a determining role in wheat yield. Insufficient soil moisture may diminish the positive effects of nitrogen fertilization on wheat production, while heavy rainfall or excessive irrigation could lead to nitrogen leaching, thereby adversely affecting yield (Basso et al., 2012). Soil pH was found to be a limiting factor for wheat HI in arid regions (p < 0.05). Wheat typically thrives in neutral soils, whereas arid regions often have alkaline soils. This inherent alkalinity in arid-area soils consequently constrains potential improvements in wheat harvest index.

## 5 Conclusions

This study, conducted from 2022 to 2023 in Xinjiang, a representative arid region of China, integrated two years of large-scale systematic sampling with multidimensional factors (geoclimatic conditions, agronomic management practices, and soil nutrient status). By employing Kriging interpolation, random forest analysis, structural equation modeling (SEM), and multiple linear regression, we systematically revealed the spatial distribution patterns and key driving mechanisms of wheat harvest index (HI) under both irrigated and rainfed conditions in arid regions. The principal findings are as follows: Wheat HI in the arid regions showed significant spatial heterogeneity, with an overall distribution pattern of “high in the center, low in the surroundings, high in the north and low in the south”. Both irrigated and rainfed wheat showed the characteristics of “high in the north and low in the south”, but there were obvious differences, and the HI of irrigated wheat was significantly higher than that of rainfed conditions (0.43-0.67). The overall wheat driver importance ranking intensity in the arid regions was in the following order: soil nutrient factor, agronomic management factor, and geo-climatic factor; for irrigated wheat it was geo-climatic factor, soil nutrient factor, and agronomic management factor; while for rainfed wheat it was soil nutrient factor, geo-climatic factor, and agronomic management factor. Comprehensive multi-method analyses (correlation analysis, random forest, and structural equation modeling) indicated that H, TP, AP, TN, and SWC were the key drivers constituting the HI of wheat in the dry regions. Irrigation can increase HI, yield and AGB compared with rainfed, and optimized phosphorus fertilizer management can also increase HI. This study provides a theoretical basis and practical guidance for the synergistic multi-objective approach of “yield increase-irrigation-sustainability” in arid regions wheat production. Future research should focus on the dynamic response mechanism and adaptive regulation of HI in the context of climate change, and more wider studies will be included to appreciate the variability of HI in different environment in the arid regions.

## Data Availability

The raw data supporting the conclusions of this article will be made available by the authors, without undue reservation.
