# Supplementary material for: Spatial patterns and key driving factors of wheat harvest index under irrigation and rainfed conditions in arid regions
Source: Front Plant Sci. 2025 Jun 9;16:1614204. doi: 10.3389/fpls.2025.1614204 (PMC12183048; doi:10.3389/fpls.2025.1614204)
Supplement: Supplementary file 1 [file SupplementaryFile1.docx]

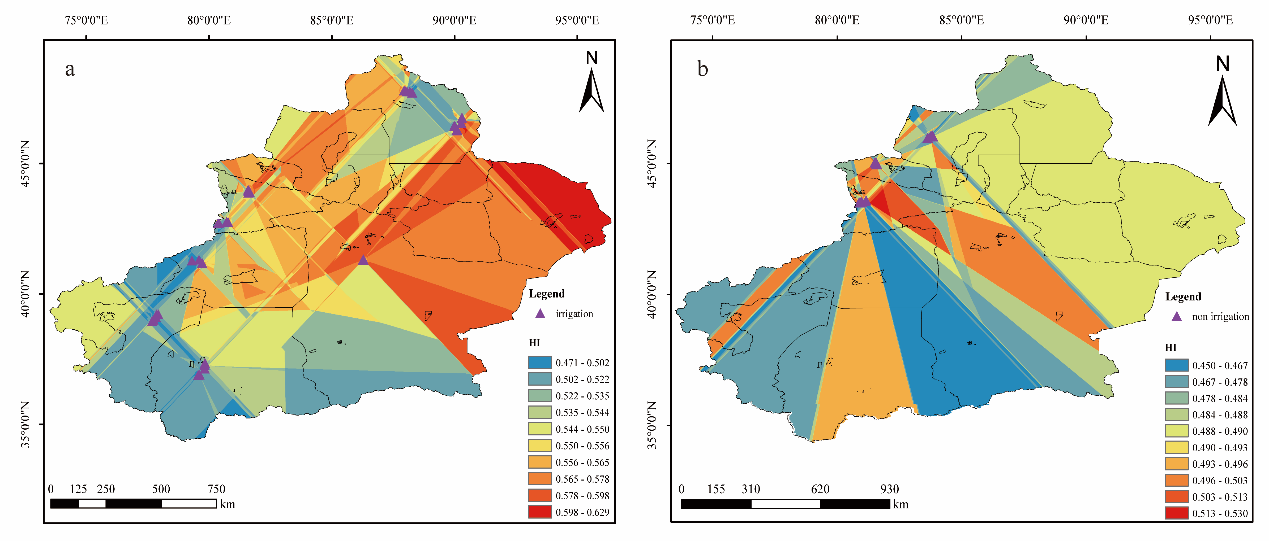


Fig. 1 | HI spatial patterns under different water regimes: (a) irrigated, (b) rainfed wheat

**Table 1. Parameters of semivariogram models for irrigation wheat HI in arid regions**

|  | Nuggget  C_0_ | Sill  C+C_0_ | RANGE  A_0_(Km) | Proportion  (C_0_/C+C_0_) | Theoretical model | CV | ME | RMSE |
| --- | --- | --- | --- | --- | --- | --- | --- | --- |
| Harvest index | 0.004282 | 0.008708 | 7.0802 | 49.17% | Log | 2.5% | 0.0005 | 0.0424 |

**Note**: Log: Logarithmic model，**CV**: Coefficient of variation

**Table 2. Parameters of semivariogram models for rainfed wheat HI in arid regions**

|  | Nuggget  C_0_ | Sill  C+C_0_ | RANGE  A_0_(Km) | Proportion  (C_0_/C+C_0_) | Theoretical model | CV | ME | RMSE |
| --- | --- | --- | --- | --- | --- | --- | --- | --- |
| Harvest index | 0.000158 | 0.000524 | 0.618 | 30.15% | Gaussian | 0.61% | 0.0161 | 0.0237 |

**Note:** Gaussian: Gaussian model，**CV**：Combined with coefficient of variation
